# Editorial: The Psychophysiology of Action

**DOI:** 10.3389/fpsyg.2019.01266

**Published:** 2019-05-31

**Authors:** Sven Hoffmann, Christian Beste, Markus Raab

**Affiliations:** ^1^Institute of Psychology, German Sport University Cologne, Cologne, Germany; ^2^Dresden University of Technology, Dresden, Germany; ^3^Universitätsklinikum Carl Gustav Carus, Dresden, Germany; ^4^London South Bank University, London, United Kingdom

**Keywords:** performance, movement science, sports, neuroscience, perspective, emotions, motor performance, voluntary action

What is action? What processes are involved in initiating, guiding, and evaluating the outcomes of action? Different research disciplines have dealt with these questions, and a huge amount of empirical and theoretical work has been conducted so far. However, only a few attempts have been made to integrate the different perspectives. We think it is time to bring together the fields of psychology, neuroscience, and movement/performance science, to stimulate the in-depth exchange of ideas and advance the “psychophysiology of action” as a topic of interest, since psychophysiology and its methods provide a bridge to connect these areas. Further, we assume that to investigate actions in dynamical environments, corresponding measures are necessary that reflect the dynamics of movements; also, multivariate measures should be considered and the dynamics should be reflected in corresponding statistical parameters. Thus, the goal is to bring together theoretical and empirical research from several disciplines to foster the exchange of ideas and methods in an effort to investigate the dynamical role of movement in cognition. Therefore, we invited authors to submit research articles targeting the understanding of action across theories and disciplines.

Hoffmann et al. suggest that to study the psychophysiology of action, it is necessary to consider multiple methodological challenges. The authors describe selected theoretical accounts of how internal and external information processes interact. They suggest that research on the dynamics of action should (a) consider the dynamics of movement, (b) make use of multivariate measures, and (c) employ dynamic statistical parameters accounting. The articles presented within this research topic are diverse with respect to these three dimensions and show that each research area or research discipline touches on at least one dimension.

Indeed, if considering a multidimensional account of action, one has to think about biological motion and how this affects the perception of emotional states. Bachmann et al. show in their review that most brain regions display increased reactivity to emotional body movements in general and that some structures are related selectively to negative valence.

In addition, emotions and the perception thereof play a crucial role in the psychophysiology of action. Masaki et al. show that anxiety in sports situations where participants are evaluated is connected to feedback processing, as measured by oscillations in electroencephalograms (EEGs). They found that theta was increased for high-anxiety groups compared to low-anxiety groups, and delta was higher for a high-anxiety group, but only in an evaluation condition.

In everyday life, people find that music affects their emotions, and thus action. Kuan et al. utilized several measures (e.g., galvanic skin response and heart rate) to investigate the effect of relaxing and arousing music during imagery on dart-throwing performance. Overall, they found positive effects of relaxing music on several parameters.

The study by Cartaud et al. shows that measures of peripheral physiology might be useful for investigating social interactions: They found that interpersonal distances are relevant for effective social interactions. They utilized electrodermal activity as an indicator of emotional responses in a paradigm manipulating facial expressions (via point-light displays) and their respective peri-/extrapersonal positions. Their findings suggest that peripersonal action space and interpersonal social space are sensitive to the emotional valence of a confederate such that the personal comfort distance is affected.

Munzert and Krüger remind us of a crucial historical contribution to action research in their review: Already in the 1930s Edmund Jacobson demonstrated, as a precursor of motor imagery, that peripheral physiological effects rely on task-specific instructions. This historical perspective highlights the relevance of integrating peripheral and central mechanisms related to actions.

Performance is not modulated only by relaxation techniques or music. A recent neuroscience technique, transcutaneous vagus nerve stimulation (tVNS), might have an effect on core mechanisms of action control. This points to a close connection between peripheral and the central nervous system. Jongkees et al. investigate whether key transmitter systems related to action control—gamma-aminobutyric acid and noradrenaline—are linked to tVNS. In a serial reaction time task, they found that tVNS enhanced response selection processes.

Vidal et al. outline that the activity of motor areas seems to depend on the nature of the executed movement as well as on the cognitive context of these movements. In their review, they describe how different classes of reaction time tasks allow specifying the nature and the dynamics of motor areas' activation in different cognitive contexts. Further, the authors describe experimental results obtained from high temporal resolution methods such as EEG during voluntary action.

With respect to voluntary action, the question is how such actions are controlled. In this context, errors are of particular interest since error processing is crucial for motor learning. Joch et al. investigate how this error monitoring is involved in motor control. In a complex motor task, they show that the error negativity (N_e_), a key correlate of action control, is modulated by the availability of different sensory signals in a semi-virtual throwing task. Their study suggests that in tasks where visual targets indicate motor performance, visual signals might be weighted more strongly than proprioceptive signals.

Maruo et al. demonstrate that in addition to the N_e_, another correlate of action control, the error positivity (P_e_), is a key correlate of error evaluation. The P_e_ is linked to monitoring one's own emotional state, such as anxiety. They show that these correlates differentiate between different types of sports; specifically, long-distance runners, and sprinters differed with respect to the N_e_ in an inverse manner: With increasing levels of competitive anxiety, the sprinters' N_e_ amplitude decreased, whereas the long-distance runners' N_e_ increased. This finding suggests that the two groups utilize their internal error-monitoring function differently.

Going back to the base of motor control, the study by Rönnquist et al. targets the questions “if” and “how” timing training might influence movement performance in athletes. They test the effect of synchronized metronome training on sensorimotor timing ability and whether that timing is related to lower limb movement planning, precision performance, and kinematics.

The study by di Fronso et al. puts endurance into the context of action control and reveals that focusing attention on core components of actions improves functional connectivity among specific brain areas and leads to enhanced performance.

The study by Betti et al. deals with a more basic question related to reach-to-grasp movements: Are corticospinal activity, kinematics, and electromyography associated with the planning and execution of prehensile actions toward either a small or a large object? By inducing motor-evoked potentials with transcranial magnetic stimulation and using several other measures, they found evidence that the index finger is involved in differential motor preparation for different types of grasps and whole-hand prehensile actions.

Fritz et al. contribute an interesting study on endurance. They show that combined musical agency experience and physical exercise can reduce pain perception, hypothetically via some endogenous opioid mechanism. This suggests that the combination of musical agency experience and physical exercise could be utilized in rehabilitation therapies where sometimes the physical treatment may be painful.

The research presented here reflects the diversity of disciplines involved in the psychophysiology of action, as well as their theories and empirical findings. Yet this impressive collection is but a stepping stone on the path to a full understanding of the topic. We hope that this multidisciplinary approach will motivate interested readers to go beyond their own discipline, that is, to go beyond their own theoretical and methodological borders: Together, we may arrive at a “psychophysiology of action.”

## Author Contributions

SH wrote the manuscript. CB and MR co-authored and edited the manuscript.

### Conflict of Interest Statement

The authors declare that the research was conducted in the absence of any commercial or financial relationships that could be construed as a potential conflict of interest.

